# Prognostic Significance of CD56 Antigen Expression in Patients with *De Novo* Non-M3 Acute Myeloid Leukemia

**DOI:** 10.1155/2021/1929357

**Published:** 2021-04-08

**Authors:** Yanni Sun, Jia Wan, Qiuyue Song, Chengxin Luo, Xi Li, Yanrong Luo, Xiangtao Huang, Ruiheng Ding, Hui Li, Yu Hou, Yongxiu Huang, Mingling Xie, Zhen Huang, Yali Zhang, Yanni Ma, Guixian Wu, Shuangnian Xu, Jieping Chen

**Affiliations:** ^1^Center for Hematology, Southwest Hospital, Army Medical University, 400038, China; ^2^College of Military Preventive Medicine, Army Medical University, Chongqing 400038, China

## Abstract

Acute myeloid leukemia (AML) is a heterogeneous group of disorders with distinct characteristics and prognoses. Although cytogenetic changes and gene mutations are associated with AML prognosis, there is a need to identify further factors. CD56 is considered a prognostic factor for AML, which is abnormally expressed in leukemia cells. However, a clear consensus for this surface molecule is lacking, which has prompted us to investigate its prognostic significance. Bone marrow samples of *de novo* non-M3 AML were collected to detect CD56 expression using multiparameter flow cytometry (FCM). As a result, the CD56 expression in *de novo* non-M3 AML was found to be significantly higher than that in acute lymphoma leukemia (ALL, *P* = 0.017) and healthy controls (*P* = 0.02). The X-Tile program produced a CD56 cutoff point at a relative expression level of 24.62%. Based on this cutoff point, high CD56 expression was observed in 29.21% of *de novo* non-M3 AML patients. CD56*-high* patients had a poor overall survival (OS, *P* = 0.015) compared to CD56*-low* patients. Bone marrow transplantation (BMT) improved OS (*P* = 0.004), but a poor genetic risk was associated with an inferior OS (*P* = 0.002). Compared with CD56*-low* patients, CD56*-high* patients had lower peripheral blood platelet (PLT) counts (*P* = 0.010). Our research confirmed that high CD56 expression is associated with adverse clinical outcomes in *de novo* non-M3 AML patients, indicating that CD56 could be used as a prognostic marker for a more precise stratification of *de novo* non-M3 AML patients.

## 1. Introduction

Acute myeloid leukemia (AML) is a heterogeneous hematopoietic malignancy caused by the malignant proliferation of immature myeloid cells. Malignant cells proliferate dramatically in the bone marrow and peripheral blood, leading to a series of serious clinical syndromes, such as anemia, hemorrhage, and infection. AML is fatal if it is not treated in time [1].

Clinically, AML can be divided into eight subtypes, from M0 to M7, according to the French-American-British (FAB) classification of AML. It can also be classified into four major types, according to the 2016 WHO classification [2]. Currently, patients with AML respond variably to treatment as a result of clinical and genetic heterogeneity [3–5]. Recent advances in genetic markers have stratified patients with AML into favorable, intermediate, and adverse ELN risk categories. However, this stratification cannot fulfill all clinical situations, and patients with refractory AML are found across all risk groups [6]. This suggests that certain nongenetic factors also play an important role in the prognosis of AML, indicating that AML patients require a more precise prognostic stratification.

Various clinical and biological parameters have been reported to affect the prognosis of AML. Previous studies have shown a significant association between immunophenotype and treatment response or survival [7, 8]. New immunological biomarkers have also recently emerged, providing many new therapeutic targets [9, 10].

CD56, also known as neural cell adhesion molecule 1 (NCAM1), is a 180 kD glycoprotein that mediates hematopoietic cell adhesion and is involved in cytotoxicity [11]. Located in a breakpoint region on chromosome 11, CD56 can be abnormally expressed in leukemia cells [12]. Some researchers have reported that CD56 overexpression may adversely affect AML treatment response and survival [13, 14], while others have reported conflicting results [15, 16]. Therefore, there is a need to determine whether CD56 is a suitable biomarker for predicting the prognosis of AML.

## 2. Materials and Methods

### 2.1. Patient Selection and General Information

This single-center retrospective study included 89 consecutive patients with a diagnosis of *de novo* non-M3 AML between March 1, 2016 and March 1, 2019. Acute promyelocytic leukemia (APL, also known as AML-M3) is a special type of AML characterized by the PML-RAR*α* fusion gene generated by the t (15; 17) (q22; q21) chromosomal translocation. With the widespread use of all-trans retinoic acid (ATRA) and arsenic trioxide (ATO), at least 80% of M3 patients can be cured [17–20]. Therefore, APL was excluded from our study. Patients diagnosed with therapy-related myeloid neoplasms (t-AML), AML derived from myelodysplastic syndrome (MDS), or secondary to other malignancies (AHD AML) were also excluded because of the complexity of these clinical conditions.

CD56 expression was detected in the bone marrow using multiparameter flow cytometry (FCM) before and after treatment. In addition, markers of leukemic cells in acute lymphoma leukemia (ALL) patients and myeloid lineage cells in healthy people were also evaluated before the administration of the relevant treatment for use as controls. The AML and control groups were matched by age and sex. All leukemia patients in this study were diagnosed based on the 2016 World Health Organization (WHO) classification of myeloid neoplasms and acute leukemia and treated at the Center for Hematology of Southwest Hospital affiliated with Army Medical University. Healthy controls were included based on normal bone marrow morphological examination. All studies were conducted in accordance with the Declaration of Helsinki (1964) and approved by the Ethics Committee of the Southwest Hospital. Informed consent was obtained from all the patients.

### 2.2. Treatment

Patients received induction chemotherapy consisting of anthracyclines from day 1 to 3, followed by the administration of cytarabine via intravenous infusion for 7 days (3+7 regimen) using the dose suggested by the National Comprehensive Cancer Network (NCCN)guidelines [21]. Consolidation chemotherapy consisting an intermediate dose of cytarabine (1–2 g/m^2^, q12h for three days) was administered to patients with low and intermediate risk who achieved complete remission (CR). Patients in the genetic high-risk group received either a high dose of cytarabine (3 g/m^2^, q12h for three days) or allogeneic hematopoietic stem cell transplantation. Patients also received supportive care for transfusion or appropriate antibiotics according to their disease status.

### 2.3. Flow Cytometric Analysis

Bone marrow samples were collected before and after treatment and stored in 2 mL EDTA anticoagulant tubes at 4°C for 72 h for evaluation by flow cytometry. For the immunofluorescence assay, mouse IgG conjugated to fluorescein isothiocyanate (FITC), phycoerythrin (PE), peridinin-chlorophyll-protein complex (PerCP), and allophycocyanin (APC) were used as negative controls. Leukemic blasts were immunostained with fluorescent-labeled monoclonal antibodies. The main antihuman antibodies used were as follows: FITC-, PE-, PerCP-, or APC-conjugated CD56, HLA-DR, CD34, CD117, cMPO, cCD3, cCD79a, and CD45 (Becton Dickinson, USA). Antibodies were added into the cell suspension and incubated in the dark for 15 min at room temperature. Then, red blood cells were lysed using red blood cell lysate (Becton Dickinson, USA), and the remaining cells were re-suspended in 1× phosphate buffer solution (PBS) and identified by flow cytometry (FACSCalibur, Becton Dickinson, USA). At least 3 × 10^4^ cells were acquired. Plots were created based on the CD45 fluorescence intensity and side scattered (SSC) light. Leukemic blasts were gated and judged based on the expression pattern for each marker, according to the definition of myeloid leukemia cells established in 2017 ELN [6]. The relative expression of CD56 in the leukemia blasts was analyzed using CellQuest Pro software (Becton Dickinson, USA).

### 2.4. Follow-Up Study

All observations were performed according to the International Working Group Consensus Guidelines for diagnosis, standardization of response criteria, treatment outcomes, and reporting standards for therapeutic trials in acute myeloid leukemia [22]. The morphological complete remission (CR) criteria were defined as less than 5% blasts in the bone marrow. Overall survival (OS) was defined as the time from enrollment to death (for any reason) or the final follow-up date (censored). In patients who achieved CR after treatment, disease-free survival (DFS) was calculated from the date of morphological remission until the recurrence of disease or death.

### 2.5. Data Analysis

The cutoff point of CD56 was calculated using X-Tile software based on OS [23]. Patients were divided into CD56*-high* and CD56*-low* groups, according to the cutoff point. OS and DFS were assessed using the Kaplan–Meier method, using the log-rank test to compare the curves of the different groups. Normally distributed data were compared using the Chi-squared test or independent-sample *t*-test. Other clinical characteristics or outcomes were compared using the Mann–Whitney *U* test. Univariate or multivariate Cox proportional hazards models were used to determine the risk factors. Multivariate analysis mainly included the potential prognostic factors generated from univariate analysis. Statistical analysis and graphics were performed using IBM® SPSS® Statistics 22.0 and GraphPad Prism® version 6.0.

## 3. Results

### 3.1. The Overexpression of CD56 in *De Novo* Non-M3 AML Patients

**Figure 1:**
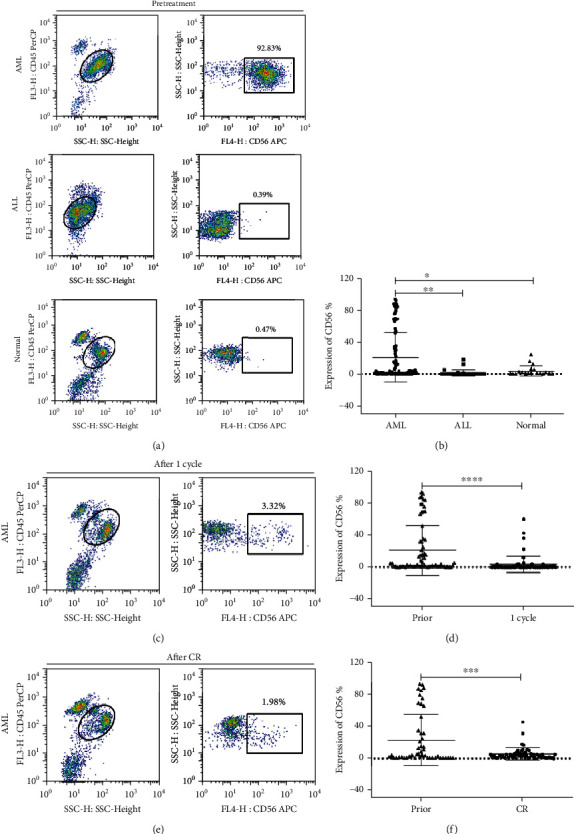
CD56 overexpression in *de novo* non-M3 AML patients. (a, b) Flow cytometry plots of the CD56 expression in *de novo* non-M3 AML, ALL, and healthy (normal) patients before treatment. Scatter plot showing a higher intensity of the CD56 expression in *de novo* non-M3 AML compared with ALL (*P* = 0.017) and normal controls (*P* = 0.02). (c, d) Markedly decreased the CD56 expression in d*e novo* non-M3 AML patients after the first induction treatment (1 cycle) (*P* < 0.001). (e, f) After remission (CR), CD56 still remained in the very low expression level (*P* < 0.001).

We detected and evaluated the expression of CD56 in 135 patients before treatment, including 89 *de novo* non-M3 AML, 27 ALL, and 19 healthy individuals, using flow cytometry. As shown in Figures 1(a) and 1(b), the independent-sample *t*-test indicated that *de novo* non-M3 AML patients showed a higher intensity of the CD56 expression compared with ALL (*P* = 0.017) or the healthy controls (normal BM) (*P* = 0.02). This indicates that the CD56 antigen is more likely to be highly expressed in AML cells. Then, the levels of CD56 expression after one cycle of induction therapy in AML patients were evaluated. As a result, the mean expression value of this antigen was found to decrease markedly (*P* < 0.001), according to paired samples test, as shown in Figures 1(c) and 1(d). In the patients that achieved complete remission, the CD56 expression remained very low compared with the expression before treatment (*P* < 0.001), as verified by the paired sample test (Figures 1(e) and 1(f)).

### 3.2. Clinical Characteristics of *De Novo* Non-M3 AML Patients

The clinical characteristics of the 89 AML patients are shown in Table 1. Their mean age was 39 years, including 42 (47.19%) men and 47 (52.81%) women. Most patients were classified into the intermediate genetic risk (ELN risk) group (61, 68.54%). In the favorable group, the genetic abnormality was t (8; 21) (q22; q22.1) or RUNX1-RUNX1T1. Patients with complex karyotypes were the main genetic phenotype in the adverse group, and cytogenetic abnormalities not classified as favorable or adverse were the most common in the intermediate group (Table S1). Fifteen (16.85%) patients underwent bone marrow transplantation (BMT).

The X-Tile program produced a CD56 cutoff point at a relative expression level of 24.62%. Therefore, the patients were divided into CD56-*high* (29.21%) and CD56*-low* (70.79%) groups according to the relative level of CD56 expression compared to the cutoff point. No significant differences in age, sex, blasts, peripheral white blood cell (WBC) count, hemoglobin (HB), and FAB classification were observed between the two groups. However, the peripheral blood platelet (PLT) count in the CD56*-high* group was lower than in the CD56*-low* group (21.5 × 10^9^/L versus 32.5 × 10^9^/L, *P* = 0.010). This suggests that the clinical symptoms of the CD56*-high* group were more serious. Genetic risk stratification was evaluated by gene mutation and chromosomal abnormalities and classified into favorable, intermediate, and adverse ELN risk groups, according to the recommendations of an international expert panel, on behalf of the European LeukemiaNet (ELN) [6]. No statistically significant differences for ELN risk were observed between the two groups, indicating that both groups had the same genetic background. The proportion of patients who received BMT did not differ between the two groups.

### 3.3. Correlation of CD56 Expression with CR and RR

In our cohort, 69.33% of *de novo* non-M3 AML patients achieved complete remission (CR) after standard treatment, while 35.85% experienced disease recurrence. However, one-way analysis of variance (ANOVA) showed no significant differences in CR and relapse rate (RR) between the CD56*-high* and CD56*-low* groups (*P* = 0.053).

### 3.4. Correlation of CD56 Expression with OS and DFS

**Figure 2:**
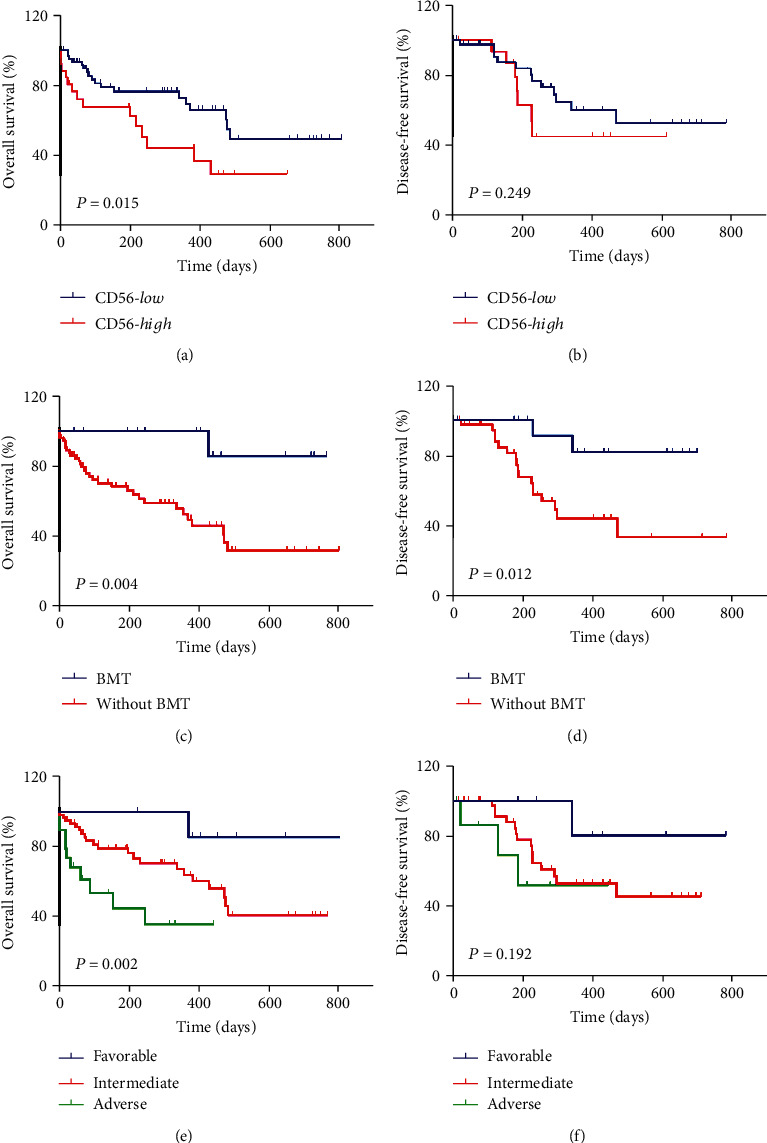
Kaplan–Meier curves of the survival times of *de novo* non-M3 AML patients. (a, b) OS and DFS of patients in CD56-*high* (*P* = 0.015) and CD56-*low* (*P* = 0.249) groups. (c, d) OS and DFS of patients administered bone marrow transplantation (BMT) (*P* = 0.004) or not (Without BMT) (*P* = 0.012). (e, f) OS and DFS of different ELN risk groups (*P* = 0.002, *P* = 0.192).

The survival outcomes of 89 AML patients in the different groups were analyzed. High levels of CD56 expression were significantly associated with a shorter OS (*P* = 0.015), according to Kaplan–Meier analysis (Figure 2(a)). The median DFS of patients with different levels of CD56 expression showed no statistical differences (*P* = 0.249), as shown in Figure 2(b).

Failure to accept BMT was significantly associated with a shorter OS (*P* = 0.004) and DFS (*P* = 0.012), as shown in Figures 2(c) and 2(d). Poor ELN risk was significantly associated with a shorter OS (*P* = 0.002) but showed no significant impact on DFS in this cohort (*P* = 0.192), as shown in Figures 2(e) and 2(f).

Table 2 presents the univariate and multivariate Cox proportional hazard models for OS. Parameters that may have an impact on survival were included in the univariate analysis process, including age as a continuous variable, ELN risk (favorable, intermediate, and adverse) as ranked data, BMT (have or not accepted BMT), and CD56 expression (CD56-*high* or CD56-*low*) as qualitative data. The univariate Cox proportional hazard model showed that ELN risk, BMT, and CD56 had a significant impact on the OS of patients with *de novo* AML. In our cohort, age did not significantly affect patient prognosis. Further multivariate analysis was conducted, and significant impact variables in the univariate analysis results were included. Using multivariate analysis, high levels of CD56 expression were found to significantly affect the OS of *de novo* non-M3 AML patients adversely, with a hazard ratio (HR) of 2.719 (*P* = 0.006), in line with the consensus that accepting BMT is an optimistic factor with an HR of 0.098 (*P* = 0.023), and poor ELN risk is an adverse prognostic factor with an HR of 3.874 (*P* < 0.001).

## 4. Discussion

ELN risk classification for AML is well established and has been extensively used as a criterion for the application of risk-adapted treatment approaches. However, clinical heterogeneity remains a commonly observed phenomenon in patients within the same genetic risk group. Therefore, this stratifying parameter needs to be supplemented with other biological factors to provide a more precise prognostic prediction to guide risk-adapted therapy.

CD56 is an isoform of neural cell adhesion molecules [24]. Because of its involvement in cell–cell interactions, regulation of cell homing, and the pattern of malignant AML cell dissemination [25–27], it may be of prognostic value for AML. Thus, in the present study, the relationship between the expression of surface antigen CD56 and the prognosis of *de novo* none-M3 AML patients was analyzed.

In our cohort, approximately one-third of *de novo* non-M3 AML patients aberrantly expressed CD56 in the bone marrow AML blasts. Patients in the CD56*-high* group were found to have a significantly worse prognosis, demonstrating a shorter OS. Meanwhile, a shorter DFS was observed in the CD56*-high* group for patients who achieved CR after treatment, showing a consistent pattern with OS.

CR and RR are both valuable parameters for assessing the response of disease to therapy. In the present study, CR or relapse after regular chemotherapy shared a similar ratio in the groups classified according to different levels of CD56 expression, indicating that there was no difference in the treatment response between the two groups. Throughout the administration of chemotherapy treatment, the levels of CD56 antigen expression in the bone marrow myeloid cells of *de novo* non-M3 AML patients were detected. As a result, the intensity of the CD56 antigen expression was found to decrease markedly alongside improved disease conditions. The inconsistency of the results between CR and relapse with survival indicate that CD56 affects the long-term survival of AML patients via mechanisms other than treatment response.

The clinical features of the AML patients in the CD56*-high* and CD56*-low* groups were analyzed, and, as a result, the two groups were found to have similar WBC counts and hemoglobin, in addition to the same composition in the FAB classification. Interestingly, patients expressing high levels of the CD56 antigen had lower PLT counts, suggesting that more attention should be paid to patients with high levels of CD56 expression for bleeding tendency. The results for genetic risk, a critical factor affecting patient outcomes, were closely in line with the consensus, which was verified in univariate and multivariate analyses. Bone marrow transplantation is known as an effective treatment for AML, yielding a high rate of curability [28, 29]. In the present study, no differences were observed in terms of genetic risk and bone marrow transplantation rate between the different groups. However, it is worth noting that the groups were similar in terms of the genetic background of the patients and the treatment approaches. According to the Cox proportional hazards model, bone marrow transplantation was a protective factor, consistent with previous reports and clinical experiences. These results indicate that high levels of the CD56 expression are an independent risk factor for adverse survival.

No correlation between CD56 expression levels and gene mutations was observed in the present study. This may be due to the small number of participants or could arise from a lack of correlation specifically in Southwest Chinese patients. To clarify this issue, more patients will be recruited in future studies to determine the correlation between the CD56 expression and mutation status.

Although hematopoietic stem cell transplantation is considered a poor choice, treatment costs and graft versus host disease (GVHD) remain difficult challenges in the treatment of AML [30, 31]. More critical factors, such as the CD56 molecule, which exerts comparable roles via an unknown mechanism, remain to be discovered. Recently, researchers found that CD56 may act as a promoter to initiate drug resistance in AML patients [32]. New therapeutic strategies for the treatment of AML include small molecule targeted therapy and immunotherapy [33]. The former selectively targets specific signaling pathways but is prone to drug resistance [34]. By contrast, the treatment mechanisms of the latter include complex antibodies and cellular immunity, showing promising results as an AML treatment [35–37]. Currently, advances in the field of immunotherapy are in full swing. With the aim of improving treatment effects and reducing drug resistance, CD56 is a prospective target. Studies have shown that the antibody-drug conjugate m906 PBD induces cell death in CD56-positive neuroblastoma cell lines [38]. In addition, CAR-T cells against CD123 antigens show good potential [39, 40]. However, more appropriate antigens and the corresponding protocols are yet to be discovered, with prospects including antibody or cellular immunity, such as CAR-T cell therapy for CD56 in AML.

Taken together, our findings indicate that high levels of CD56 expression are a factor for poor prognosis in *de novo* non-M3 AML patients. These results suggest that CD56 may be a potential target for targeted AML therapy, particularly in patients with CD56 overexpression. However, due to the present study's shortcomings in terms of follow-up time and censored cases, further clinical and experimental evidence will be needed to verify these results.

## 5. Conclusion

The findings present in this study confirmed that high levels of CD56 expression are associated with adverse clinical outcomes in AML patients, indicating that CD56 could be considered as a prognostic marker for a more precise stratification of non-M3 AML patients. Further comprehensive studies will be needed to verify these results and elucidate the mechanisms underlying the action of this marker in potential therapeutic strategies for AML.

## Figures and Tables

**Table 1:** Clinical characteristics of de novo non-M3 AML patients according to the CD56 expression.

Variables	All patients, *n* (%)	CD56-low, *n* (%)	CD56-high, *n* (%)	*P* value
No. of patients	89 (100)	63 (70.79)	26 (29.21)	
Sex				
Male	42 (47.19)	24 (38.10)	18 (69.23)	0.343
Female	47 (52.81)	39 (61.90)	8 (30.77)	
Age, years				0.143
Mean (min-max)	39 (12~64)	41 (13~64)	36 (12~61)	
BM blasts (%)				0.244
Median	67.70	63.39	73.20	
Range	15.57~97.25	15.57~97.25	33.50~95.00	
WBC (×10^9^/L)				0.632
Mean (min-max)	39.76 (0.59~365.67)	41.55 (0.95~365.67)	35.19 (1.28~148.90)	
HB (g/dL)				0.336
Mean (min-max)	76.03 (1.78~149.00)	77.71 (1.78~149.00)	71.65 (37~107)	
PLT (×10^9^/L)				0.010
Median	27.00	32.5	21.5	
Range	3~301	3~301	5~83	
FAB classification				0.471
M1	9 (10.11)	5 (7.94)	4 (15.38)	
M2	27 (30.34)	20 (31.75)	7 (26.92)	
M4	28 (31.46)	24 (38.10)	4 (15.38)	
M5	15 (16.85)	11 (17.46)	4 (15.38)	
Unclassified	5 (5.62)	3 (4.76)	2 (7.69)	
Cytogenetic risk				0.274
Favorable	9 (10.11)	5 (7.94)	4 (15.38)	
Intermediate	61 (68.54)	43 (68.25)	18 (69.23)	
Adverse	19 (21.35)	15 (23.81)	4 (15.38)	
Received BMT				1.000
BMT	15 (16.85)	11 (17.46)	4 (15.38)	
Without BMT	74 (83.15)	52 (82.54)	22 (84.62)	

BM: bone marrow; WBC: white blood cell; Hb: hemoglobin; PLT: platelet; BMT: bone marrow transplantation.

**Table 2:** Univariate and multivariate analyses of factors correlating to overall survival.

Variables	Univariate analysis		Multivariate analysis	
	HR (95% CI)	*P* value	HR (95% CI)	*P* value
Age	1.017 (0.989, 1.045)	0.236	/	/
Cytogenetic risk	3.190 (1.623, 2.269)	0.002	3.874 (1.901, 7.878)	<0.001
Received BMT	0.093 (0.013, 0.683)	0.004	0.098 (0.013, 0.724)	0.023
High CD56 expression	2.323 (1.151, 4.688)	0.015	2.719 (1.339, 5.520)	0.006

HR: hazard ratio; BMT: bone marrow transplantation; “/”: no significance in multivariate forward logistic regression analysis.

## Data Availability

The datasets used or analyzed during the current study are available from the corresponding author on reasonable request.

## Supplementary Materials

Supplementary Materials Table S1 provides the genetic characteristics of *de novo* non-M3 AML patients arranged by 2017 ELN risk stratification.
