# Supplementary material for: Prognostic Significance of CD56 Antigen Expression in Patients with De Novo Non-M3 Acute Myeloid Leukemia
Source: Biomed Res Int. 2021 Apr 8;2021:1929357. doi: 10.1155/2021/1929357 (PMC8049794; doi:10.1155/2021/1929357)
Supplement: Supplementary Materials — Table S1 provides the genetic characteristics of de novo non-M3 AML patients arranged by 2017 ELN risk stratification. [file 1929357.f1.docx]

Table S1. Genetic characteristics of de novo non-M3 AML patients arranged by 2017 ELN risk stratification.

|  | Genetic abnormality | n | % |  |
| --- | --- | --- | --- | --- |
| Favorable | t (8;21) (q22; q22.1); RUNX1-RUNX1T1 | 9 | 10.11 |  |
|  | CBFB-MYH11 | 1 | 1.12 |  |
| Intermediate | Mutated NPM1 and FLT3-ITD^high^ | 1 | 1.12 |  |
|  | MLLT3-KMT2A | 1 | 1.12 |  |
|  | Cytogenetic abnormalities not classified as favorable or adverse | 58 | 65.17 |  |
| Adverse | BCR-ABL1 | 1 | 1.12 |  |
|  | −7 | 1 | 1.12 |  |
|  | Complex karyotype | 10 | 11.23 |  |
|  | Wild-type NPM1 and FLT3-ITD^high^ | 5 | 5.62 |  |
|  | Mutated ASXL1 | 1 | 1.12 |  |
|  | Mutated TP53 | 1 | 1.12 | |

ELN, European LeukemiaNet.
